# Differential Susceptibilities of *Aedes aegypti* and *Aedes albopictus* from the Americas to Zika Virus

**DOI:** 10.1371/journal.pntd.0004543

**Published:** 2016-03-03

**Authors:** Thais Chouin-Carneiro, Anubis Vega-Rua, Marie Vazeille, André Yebakima, Romain Girod, Daniella Goindin, Myrielle Dupont-Rouzeyrol, Ricardo Lourenço-de-Oliveira, Anna-Bella Failloux

**Affiliations:** 1 Instituto Oswaldo Cruz, Rio de Janeiro, Brazil; 2 Institut Pasteur, Arboviruses and Insect Vectors, Paris, France; 3 Centre de Démoustication/Collectivité Territoriale de La Martinique, Fort-de-France, Martinique; 4 Institut Pasteur of French Guiana, Unit of Medical Entomology, Cayenne, French Guiana; 5 Institut Pasteur of Guadeloupe, Laboratory of Medical Entomology, Environment and Health, Les Abymes, Guadeloupe; 6 Institut Pasteur of New Caledonia, Dengue and Arboviruses Research and Expertise Unit, Nouméa, New Caledonia; United States Army Medical Research Institute of Infectious Diseases, UNITED STATES

## Abstract

**Background:**

Since the major outbreak in 2007 in the Yap Island, Zika virus (ZIKV) causing dengue-like syndromes has affected multiple islands of the South Pacific region. In May 2015, the virus was detected in Brazil and then spread through South and Central America. In December 2015, ZIKV was detected in French Guiana and Martinique. The aim of the study was to evaluate the vector competence of the mosquito spp. *Aedes aegypti* and *Aedes albopictus* from the Caribbean (Martinique, Guadeloupe), North America (southern United States), South America (Brazil, French Guiana) for the currently circulating Asian genotype of ZIKV isolated from a patient in April 2014 in New Caledonia.

**Methodology/Principal Findings:**

Mosquitoes were orally exposed to an Asian genotype of ZIKV (NC-2014-5132). Upon exposure, engorged mosquitoes were maintained at 28°±1°C, a 16h:8h light:dark cycle and 80% humidity. 25–30 mosquitoes were processed at 4, 7 and 14 days post-infection (dpi). Mosquito bodies (thorax and abdomen), heads and saliva were analyzed to measure infection, dissemination and transmission, respectively. High infection but lower disseminated infection and transmission rates were observed for both *Ae*. *aegypti* and *Ae*. *albopictus*. *Ae*. *aegypti* populations from Guadeloupe and French Guiana exhibited a higher dissemination of ZIKV than the other *Ae*. *aegypti* populations examined. Transmission of ZIKV was observed in both mosquito species at 14 dpi but at a low level.

**Conclusions/Significance:**

This study suggests that although susceptible to infection, *Ae*. *aegypti* and *Ae*. *albopictus* were unexpectedly low competent vectors for ZIKV. This may suggest that other factors such as the large naïve population for ZIKV and the high densities of human-biting mosquitoes contribute to the rapid spread of ZIKV during the current outbreak.

## Author Summary

Zika virus (ZIKV) is an emerging mosquito-borne arbovirus causing dengue-like symptoms. This virus was commonly detected in Africa and Asia. Since its emergence in Yap Island in Micronesia in 2007, ZIKV reemerged in the South Pacific region in 2013 and ultimately reached the American continent in 2015. The human biting mosquito *Aedes aegypti* and the less anthropophilic *Aedes albopictus* have been incriminated as vectors of ZIKV. Our study showed that American populations of *Ae*. *aegypti* and *Ae*. *albopictus* were able to become infected and disseminate ZIKV within the mosquito general cavity at early days (4, 7) post-infection (dpi). Nevertheless, transmission was unexpectedly low and only detected at 14 dpi. Our findings will help in designing more adapted vector control strategies and limiting the impact of a new emerging threat on human health in the Americas as did the chikungunya in 2014.

## Introduction

Zika virus (ZIKV; family *Flaviviridae*, genus *Flavivirus*) was first isolated from a sentinel rhesus monkey in the Zika forest of Uganda in 1947 [[1]]. Since then, it has emerged outside of its natural range of distribution, Africa and Asia: Yap Island (Micronesia) in 2007 [2], French Polynesia in 2013 [3], New Caledonia in 2014 [4], Easter Island in 2014 [5], the Cook Islands in 2014 [6] and more recently, northeastern Brazil in May 2015 [7, 8], the starting point of a pandemic in the Americas with 26 American countries reporting active ZIKV transmission (http://www.cdc.gov/zika/geo/active-countries.html). Although reports indicate that most infections produce mild signs and symptoms of rash, fever, arthritis or arthralgia, and conjunctivitis, recent infections have been associated with more severe disease outcomes with neurological or auto-immune complications such as Guillain-Barre syndrome [9] and microcephaly (http://www.cdc.gov/zika/pdfs/possible-association-between-zika-virus-and-microcephaly.pdf). This virus has a high potential for geographic expansion into countries where *Aedes* spp. mosquitoes are present notably *Aedes aegypti* mosquitoes.

The primary vectors of ZIKV in Africa are *Aedes* mosquitoes with reported viral isolations from *Ae*. *africanus* and *Ae*. *apicoargenteus* [10], *Ae*. *luteocephalus* [11], *Ae*. *furcifer* and *Ae*. *taylori* [12], and *Ae*. *vittatus* [13]. The human-biting mosquito *Ae*. *aegypti* is usually considered as a laboratory-competent vector of ZIKV [14] and viral isolations were reported from the species in the wild [13, 15, 16]. However, transmission of ZIKV by African *Ae*. *aegypti* has been unexpectedly low to null [17], underlining the importance of genetic delineation of mosquito populations on vector competence [18, 19]. In addition, *Aedes albopictus* has also been shown to be an efficient laboratory vector of ZIKV [20], with viral isolations from field-collected mosquitoes [21].

This positive-sense, single-stranded RNA virus of 10,794-nt is composed of three major lineages: East African, West African, or Asian [22]. The Asian genotype is responsible for the current expansion of ZIKV in the Americas [22–24]. As the outcome of transmission depends on the specific pairing of vector and pathogen genotypes [25], we investigated the vector competence of populations of *Ae*. *aegypti* and *Ae*. *albopictus* from the Caribbean (Martinique, Guadeloupe), North America (southern United States), South America (Brazil, French Guiana) for an Asian genotype of ZIKV.

## Materials and Methods

### Ethics statement

The Institut Pasteur animal facility has received accreditation from the French Ministry of Agriculture to perform experiments on live animals in compliance with the French and European regulations on care and protection of laboratory animals. This study was approved by the Institutional Animal Care and Use Committee (IACUC) at the Institut Pasteur. No specific permits were required for the described field studies in locations which are not protected in any way and did not involve endangered or protected species.

### Mosquito populations

Seven populations of mosquitoes (5 populations of *Ae*. *aegypti* and 2 of *Ae*. *albopictus*; (Table 1) from the Caribbean (Martinique, Guadeloupe) and continental America (southern United States, French Guiana, Brazil) were collected as larvae or using ovitraps. Eggs were hatched in dechlorinated tap water and larvae were reared under controlled conditions of 150–200 larvae per 1 liter and fed with one yeast tablet renewed every 3–4 days. Adults were kept in cages at 28°±1°C with a 16h:8h light:dark cycle, 80% relative humidity, and supplied with a 10% sucrose solution. The F1-F2 generation of mosquitoes was used for infection assays except for *Ae*. *aegypti* from Orlando (> F10) and *Ae*. *albopictus* from Vero Beach (F7).

**Table 1:** Mosquito populations collected in the Caribbean and continental Americas.

Mosquito population	Collection site	Region	Country	Generation used	Mosquito species used
CAY	Cayenne, French Guiana	South America	French Guiana	F1	AE
GUA	Baie-Mahault, Guadeloupe	Caribbean	Guadeloupe	F2	AE
JUR	Jurujuba, Rio de Janeiro	South America	Brazil	F1	AL
MAR	Pointe Chaudière, Martinique	Caribbean	Martinique	F1	AE
ORL	Orlando, Florida	North America	United States	>F10	AE
TUB	Tubiacanga, Rio de Janeiro	South America	Brazil	F1	AE
VRB	Vero Beach, Florida	North America	United States	F7	AL

AE, *Aedes aegypti*; AL, *Aedes albopictus*

### Viral strain

The ZIKV strain (NC-2014-5132) was isolated from a patient in April 2014 in New Caledonia. Viral stocks were prepared after five passages of the isolate onto Vero cells maintained at 37°C; cell infection was tracked by observation of cytopathic effect (CPE). Supernatants were collected and the viral titer was estimated by serial 10-fold dilutions on Vero cells expressed in TCID_50_/mL. The virus stock was divided into 1 mL aliquots and stored at—80°C until use. Partial sequences of the NC-2014-5132 strain showed that it is phylogenetically related to the Asian genotype as are ZIKV strains circulating in the South Pacific region [26] and Brazil [7]. Indeed, based on the NS5 gene sequence, the NC-2014-5132 strain exhibited 99.4% identity with ZIKV from Brazil (Dupont-Rouzeyrol, personal communication).

### Mosquito experimental infections

Seven day-old females were fed an infectious blood-meal containing 1.4 mL of washed rabbit erythrocytes and 700 μL of viral suspension supplemented with a phagostimulant (ATP) at a final concentration of 5 mM. For each population, 4–6 boxes of 60 mosquitoes each were exposed to the ZIKV NC-2014-5132 strain. The titer of infectious blood-meals was 10^7^ TCID_50_/mL. After the infectious blood-meal, engorged females were transferred to small containers and fed with 10% sucrose in a chamber maintained at 28°±1°C, a 16h:8h light:dark cycle and 80% humidity.

### Infection, dissemination and transmission analysis

For each population, batches of 25–30 mosquitoes were analyzed at 4 and 7 days post-infection (dpi). Additionally, *Ae*. *albopictus* from Vero-Beach (VRB) and *Ae*. *aegypti* from Tubiacanga (TUB) were examined at 14 dpi. Each mosquito was processed as follows: abdomen and thorax were examined to estimate infection, head for dissemination and collected saliva for transmission. Abdomen and thorax, and head were individually ground in 300 μL of DMEM medium supplemented with 2% fetal bovine serum (FBS). Homogenates were centrifuged at 10,000 g for 5 min before titration. Saliva was collected from individual mosquitoes as described in [27]. Briefly, wings and legs of each mosquito were removed and the proboscis was inserted into a 20 μL tip containing 5 μL of FBS. After 45 min, FBS containing saliva was expelled in 45 μL of DMEM medium for titration.

Infection rate (IR) refers to the proportion of mosquitoes with infected body (abdomen and thorax) among tested mosquitoes. Disseminated infection rate (DIR) corresponds to the proportion of mosquitoes with infected head among the previously detected infected mosquitoes (i.e; abdomen/thorax positive). Transmission rate (TR) represents the proportion of mosquitoes with infectious saliva among mosquitoes with disseminated infection. Transmission efficiency (TE) represents the proportion of mosquitoes with infectious saliva among the total number of mosquitoes tested.

### Viral titration

Body and head homogenates were serially diluted and inoculated onto monolayers of Vero cells in 96-well plates. Cells were incubated for 7 days at 37°C then stained with a solution of crystal violet (0.2% in 10% formaldehyde and 20% ethanol). Presence of viral particles was assessed by detection of CPE. Saliva was titrated on monolayer of Vero cells in 6 well plates incubated 7 days under an agarose overlay. Titers of saliva were expressed as pfu (plaque-forming unit)/saliva.

### Statistical analysis

All statistical tests were conducted using the STATA software (StataCorp LP, Texas, USA). Rates were compared using Fisher’s exact test and sample distributions with the Kruskal-Wallis test. P-values>0·05 were considered non-significant.

## Results

### Similar dissemination of ZIKV in *Ae*. *aegypti* and *Ae*. *albopictus* at early dpi

**Fig 1:**
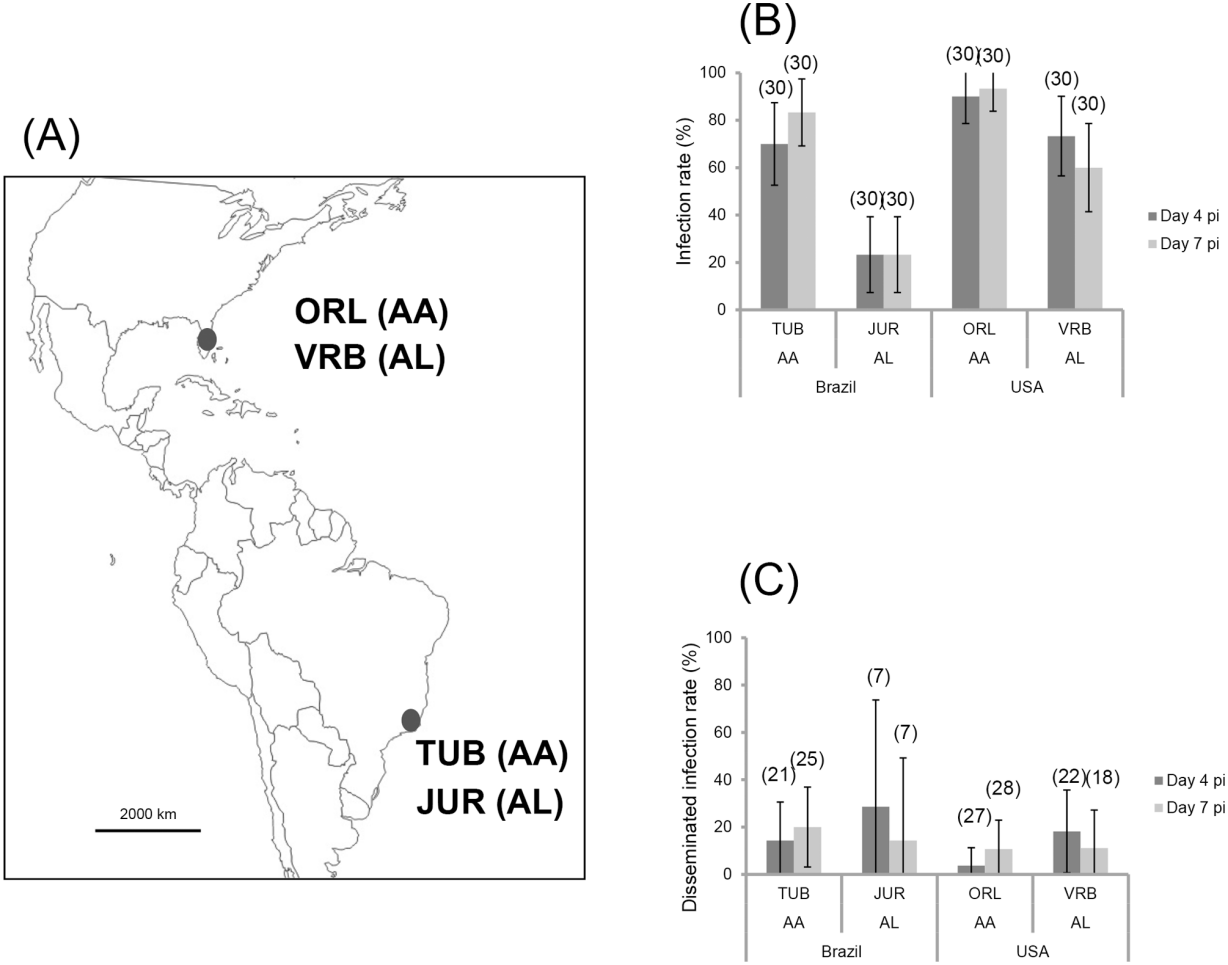
Mosquito populations (A), viral infection (B), dissemination (C) at days 4 and 7 after challenge of *Aedes aegypti* and *Aedes albopictus* from Continental America (Brazil and United States) with ZIKV provided at a titer of 10^7^ TCID_50_/mL. 30 mosquitoes were sampled each day. The error bars represent the confidence intervals (95%). The number of individuals analyzed is given in parentheses.

To define whether *Ae*. *aegypti* or *Ae*. *albopictus* were more likely to sustain a ZIKV outbreak, we analyzed the susceptibility to infection, as well as the ability of the virus to establish disseminated infection at 4 and 7 dpi in the two mosquito species collected from sites where they coexist, Rio de Janeiro (Brazil) and Florida (United States) (Fig 1A). When examining infection rates (IR) (Fig 1B), *Ae*. *aegypti* (TUB and ORL) were more likely to become infected than *Ae*. *albopictus* (JUR and VRB) (p < 0.001). Whereas the two *Ae*. *aegypti* populations examined behaved similarly (TUB *versus* ORL, p > 0.05), *Ae*. *albopictus* VRB were more infected than *Ae*. *albopictus* JUR (p = 10^−3^); infection rates at 4 and 7 dpi were lowest for *Ae*. *albopictus* JUR (N = 7 positive among 30 tested). When analyzing dissemination of infected mosquitoes (Fig 1C), disseminated infection rates (DIR) were low at 4 and 7 dpi regardless of the mosquito species and the collection site (p > 0.05). Transmission determined by detecting the presence of virus in mosquito saliva was not observed at early dpi (4 and 7) for any mosquito populations.

### *Aedes aegypti* and *Aedes albopictus* exhibit similar transmission potential for ZIKV

**Fig 2:**
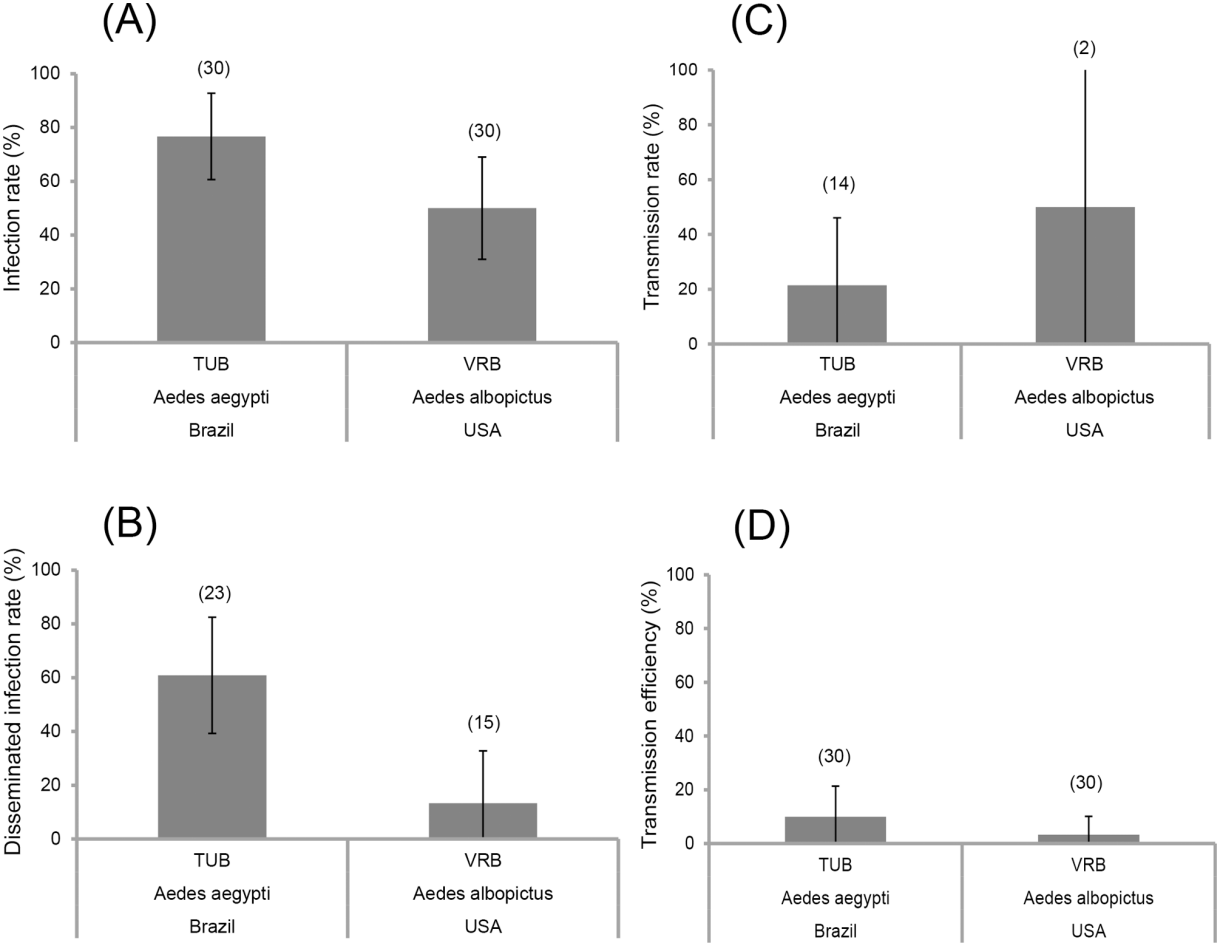
Viral infection (A), dissemination (B) and transmission (C, D) of *Aedes aegypti* TUB (Brazil) and *Aedes albopictus* VRB (United States), 14 days after oral exposure to with ZIKV. Error bars represent the confidence intervals (95%). The number of individuals analyzed is given in parentheses.

At late dpi (14), IRs, DIRs, TRs, and TEs were examined for *Ae*. *aegypti* TUB and *Ae*. *albopictus* VRB. *Ae*. *aegypti* TUB displayed higher IR and DIR than *Ae*. *albopictus* (IR: *Ae*. *aegypti* TUB: 76.7% ± 7.8 *versus Ae*. *albopictus* VRB: 50% ± 9.3, Fig 2A; DIR: *Ae*. *aegypti* TUB: 60.7% ± 10.4 *versus Ae*. *albopictus* VRB: 13.3% ± 9.1, Fig 2B). When examining the saliva of *Ae*. *aegypti* TUB and *Ae*. *albopictus* VRB at 14 dpi, TRs and viral load in saliva were higher for *Ae*. *albopictus* VRB (TR: 50% ± 50, Fig 2C; viral load: 134 ± 0 (mean ± SE), data not shown) compared to *Ae*. *aegypti* TUB (TR: 21.4% ± 11.4, Fig 2C; viral load: 18.7 ± 10.3, data not shown), even if it was not significant (P = 0.383). We should note that the number of mosquitoes examined for transmission was low despite the 30 mosquitoes initially examined. Thus we calculated the transmission efficiency showing that TEs drastically decreased to 3.3% ± 3.3 for *Ae*. *albopictus* VRB and 10% ± 5.5% for *Ae*. *aegypti* TUB (Fig 2D) suggesting that these two species were less competent to ZIKV than expected.

### *Ae*. *aegypti* in the French overseas territories of America disseminate ZIKV more efficiently

**Fig 3:**
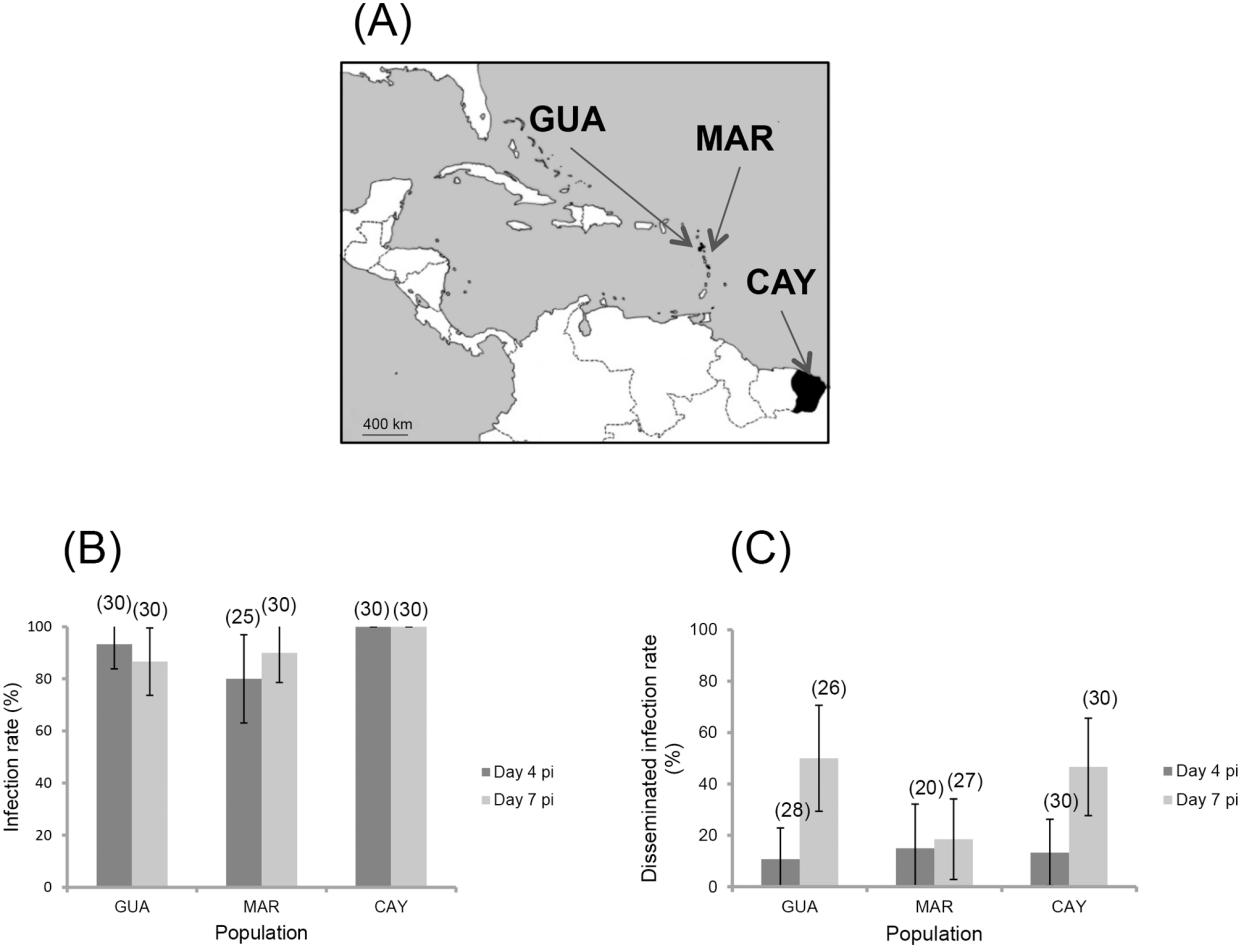
Mosquito populations (A), viral infection (B) and dissemination (C) at days 4 and 7 after oral exposure of *Aedes aegypti* from the French overseas territories of America (French Guiana, Guadeloupe and Martinique) to ZIKV provided at a titer of 10^7^ TCID_50_/mL. 25–30 mosquitoes were sampled each day. The error bars represent the confidence intervals (95%). The number of individuals analyzed is given in parentheses.

*Ae*. *aegypti* is present in French Guiana, Guadeloupe and Martinique, but where no *Ae*. *albopictus* has yet been reported (Fig 3A). We therefore determined the ability of *Ae*. *aegypti* from these territories to become infected and disseminate virus after oral exposure to ZIKV. Infection rates were high and similar regardless of dpi, and the mosquito population (Fig 3B; p > 0.05 (0.025 at 4 dpi and 0.133 at 7 dpi)). However, DIRs were reduced as compared to infection rates (Fig 3C). Viral dissemination rate, however, increased significantly with dpi except for *Ae*. *aegypti* MAR. No viral transmission was observed at 4 and 7 dpi.

When comparing all five *Ae*. *aegypti* populations at 7 dpi, DIRs were significantly different (P = 0.002) and two homogeneous groups could be distinguished: *Ae*. *aegypti* from Guadeloupe and French Guiana (P = 0.803) with higher DIRs compared to *Ae*. *aegypti* from Martinique, USA and Brazil (P = 0.609).

## Discussion

Zika virus has recently started to spread outside its natural range of distribution. After the South Pacific islands (Yap island, French Polynesia, New Caledonia, Cook islands, Easter island; [28]]), ZIKV was detected in the South American continent: Brazil in May 2015 [7], and since, 26 American countries (http://www.cdc.gov/zika/geo/active-countries.html). The first autochthonous cases were recently recorded in Martinique and French Guiana where the mosquito *Ae*. *aegypti* was assumed to be the unique vector. Our study showed that the Asian genotype of ZIKV infected and was disseminated by the vectors *Ae*. *aegypti* and *Ae*. *albopictus* collected in the Caribbean and continental America. Furthermore, we showed that *Ae*. *aegypti* from Rio de Janeiro in Brazil and *Ae*. *albopictus* from Vero Beach in the United States were able to transmit ZIKV at 14 dpi. Although susceptible to infection, these populations were unexpectedly low competent vectors for ZIKV.

After the emergence of chikungunya virus (CHIKV) from East Africa [29] followed by its worldwide expansion and establishment in the Americas of the Asian lineage since October 2013 [30], ZIKV became a second example of emergence of a vector-borne disease threatening a new continent. Both viruses originated in Africa where they circulate in an enzootic cycle involving non-human primates and a wide variety of zoophilic mosquitoes [17, 31]. Human outbreaks due to CHIKV involve anthropophilic vectors such as *Ae*. *aegypti* and to a lesser extent, *Ae*. *albopictus*. This latter species has been shown to be capable of transmitting at least 26 arboviruses in the laboratory and its implication as a main vector became a reality with the recent CHIKV pandemic [32]. *Ae*. *albopictus* transmits preferentially a CHIKV variant presenting an amino-acid change in an envelope glycoprotein [33, 34]. This viral variant was selected after passing through the midgut barrier, the first step in mosquito infection [35]. We showed that *Ae*. *albopictus* VRB restrained ZIKV dissemination highlighting the importance of barriers such as the midgut in *Ae*. *albopictus* mosquitoes. The examination of ZIKV infection in *Ae*. *aegypti* TUB underlined the significant role of salivary glands in transmission. Therefore, the specific role of salivary glands on ZIKV transmission by *Ae*. *aegypti* and the passage of the virus in this mosquito compartment should be explored more in detail as it has been done with CHIKV in *Ae*. *albopictus* [36]. However, the proportion of mosquitoes capable of transmitting ZIKV on the total number of tested mosquitoes, was unexpectedly low suggesting that these two species were poorly competent to ZIKV.

We also demonstrated that *Ae*. *albopictus* from Florida was at least two times more susceptible to ZIKV infection than *Ae*. *albopictus* collected in Rio de Janeiro, underlining differences depending on the mosquito population described under genotype-by-genotype (G x G) interactions where the outcome of infection depends on the specific pairing of vector and pathogen genotypes [37]. Additionally, the two *Ae*. *albopictus* populations examined from the Americas exhibited lower susceptibilities to ZIKV than *Ae*. *albopictus* from tropical Asia (i.e. Singapore) [20].

Zika disease can be confused with dengue fever and chikungunya fever, all transmitted by *Ae*. *aegypti* and *Ae*. *albopictus*. Viremia in patients was lower when infected with ZIKV, i.e. 10^3^−10^6^ RNA copies/mL [23] compared to viremia for dengue virus (DENV) (10^6^−10^7^ RNA copies/mL; [38] and CHIKV (10^7^−10^9^ RNA copies/mL; [39]). We thus expected a longer extrinsic incubation period (EIP) associated with the lower viremia. EIP corresponds to the time necessary for the virus to reach the mosquito saliva after an infectious blood-meal [40]. An increase of the blood-meal viral titer has been demonstrated to decrease the length of the EIP. For other flaviviruses than ZIKV, yellow fever virus and DENV, viral particles started to be detected in salivary glands of *Ae*. *aegypti* at 10 dpi [41] and at 7–9 dpi [42, 43], respectively. With ZIKV, we showed an EIP longer than 7 days with a blood-meal at 10^7^ TCID_50_/mL to *Ae*. *aegypti* and *Ae*. *albopictus* from the Americas. [41]. Of note, artificial feeding systems usually need higher viral titers to reproduce infection rates observed when mosquitoes fed on viremic hosts [44]. Capable of inducing even higher viremia, CHIKV has been mainly associated with very shorter EIP, e.g. 2 days [27]. Therefore ZIKV does not present the same features as CHIKV in mosquito populations from the Americas with a longer EIP; this longer EIP would allow a broader window for implementation of vector control measures. Surveillance and control measures against ZIKV transmission in the Americas and more specifically, in Brazil, the starting point of the Zika outbreak, mainly use measures implemented for dengue control focused on *Ae*. *aegypti* (http://portalsaude.saude.gov.br/images/pdf/2015/dezembro/09/Microcefalia---Protocolo-de-vigil--ncia-e-resposta---vers--o-1----09dez2015-8h.pdf). However, if ZIKV is able to infect and be transmitted by other mosquito species (e.g. *Culex* spp.), their role in transmission would need to be defined to help design of more adapted vector control strategies aiming to impair the spread of the Zika outbreak in the continent.

The recent introduction of ZIKV in the Americas and its rapid spread across the continent and the Caribbean is likely attributable to the globalization of trades and travels and also the ability of local *Ae*. *aegypti* and *Ae*. *albopictus* to disseminate and then to transmit the Asian genotype of ZIKV. Contrary to the scenario with CHIKV, longer EIPs of ZIKV in the populations examined would allow implementation of more adapted vector control measures leading to improved limitation of this new emerging threat to human health in the Americas. Nevertheless, both *Ae*. *aegypti* and *Ae*. *albopictus* in the Americas do not appear to be highly efficient vectors of ZIKV, which may be balanced by the large number of susceptible humans and their close contacts with *Aedes* mosquitoes.
